# Is plasticity of synapses the mechanism of long-term memory storage?

**DOI:** 10.1038/s41539-019-0048-y

**Published:** 2019-07-02

**Authors:** Wickliffe C. Abraham, Owen D. Jones, David L. Glanzman

**Affiliations:** 1 Department of Psychology, Brain Health Research Centre, Brain Research New Zealand, University of Otago, Box 56, Dunedin, 9010 New Zealand; 2 Departments of Integrative Biology and Physiology, and Neurobiology, and the Brain Research Institute, University of California, Los Angeles, CA 90095 USA

**Keywords:** Long-term memory, Long-term potentiation

## Abstract

It has been 70 years since Donald Hebb published his formalized theory of synaptic adaptation during learning. Hebb’s seminal work foreshadowed some of the great neuroscientific discoveries of the following decades, including the discovery of long-term potentiation and other lasting forms of synaptic plasticity, and more recently the residence of memories in synaptically connected neuronal assemblies. Our understanding of the processes underlying learning and memory has been dominated by the view that synapses are the principal site of information storage in the brain. This view has received substantial support from research in several model systems, with the vast majority of studies on the topic corroborating a role for synapses in memory storage. Yet, despite the neuroscience community’s best efforts, we are still without conclusive proof that memories reside at synapses. Furthermore, an increasing number of non-synaptic mechanisms have emerged that are also capable of acting as memory substrates. In this review, we address the key findings from the synaptic plasticity literature that make these phenomena such attractive memory mechanisms. We then turn our attention to evidence that questions the reliance of memory exclusively on changes at the synapse and attempt to integrate these opposing views.

## Introduction

Ever since the discovery that nerve cells communicate with each other at their synaptic connections, the role that synapses play in learning and memory has been a matter of extensive theory and experimental investigation. Theorists, for example Hebb, Milner and Stent among others^1–3^ led the way, but as experimental models of memory formation were developed together with increasingly sophisticated methodologies, the concept that experience-dependent synaptic change is a fundamental mechanism of learning and memory retention has gained overwhelming sway in neuroscience and psychology. This concept is now described as the synaptic plasticity and memory (SPM) hypothesis.^4^ Data from manipulations of visual experience during the critical period, environment enrichments during and after development, and learning-related changes at invertebrate synapses have all pointed toward this mechanism, albeit without excluding other forms of neuronal alterations such as enhanced cell excitability.^5,6^

The experimental basis for the link between synaptic change and memory storage really gained strength when in vitro and in vivo electrophysiological recordings were able to reveal activity-dependent long-lasting changes in synaptic efficacy. These include long-term potentiation (LTP) and long-term depression (LTD), typically studied at mammalian synapses, and which are expressed as changes in both pre- and postsynaptic elements,^7,8^ as well as long-term facilitation (LTF), typically studied at invertebrate synapses, and which is also expressed pre- and postsynaptically.^9^ Moreover, the development of modern imaging technologies has allowed real-time monitoring of synaptic spine morphology changes that can accompany the electrophysiological measures of synaptic function and serve as a proxy for them. All these phenomena and their connections to memory have been reviewed extensively as noted above, and it is not our intention to discuss in detail this vast field again here. We will summarize, however, some key features and mechanisms of synaptic plasticity that support the conventional view of memory storage mechanisms, to serve as a backdrop for reviewing more recent data that question the primacy of synaptic change as a mechanism for long-term memory storage. This discourse is not meant to imply that synaptic plasticity is the only form of neural plasticity that could underlie learning and memory, some of which are discussed below. But there is little evidence that these other forms are sufficient in themselves for long-term memory storage, especially in mammalian systems. Thus, synaptic plasticity has continued to play a central role in the study of the neural mechanisms of memory and for this reason it is the focus of the present discussion.

### LTP and LTD

LTP is the most studied form of synaptic plasticity, and the one that, in mammals, is most closely linked to memory storage. Foreshadowed by Hebb’s theory,^1^ and encapsulated in the phrase “cells that fire together wire together”,^10^ LTP is studied traditionally by replacing the learning experience with high-frequency electrical stimulation of a neural pathway, or repeated pairings of presynaptic and postsynaptic cell firing. In the latter case, the exact timing between the arrival of the synaptic input and the postsynaptic action potential determines whether LTP or LTD is generated. Classically, presynaptic activity before postsynaptic activity within a temporal window generates LTP, while a reversed pairing generates LTD. However, the actual plasticity outcomes can vary widely, depending on the experimental protocols used and the brain region being studied. Regardless of the induction paradigm, recordings are made of the synaptic responses to test shocks of the presynaptic fibres to assess changes in synaptic efficacy. LTP exhibits numerous electrophysiological properties that make it an attractive candidate memory mechanism: rapid induction, long-term maintenance to varying degrees but with a demonstrated capacity for persistence up to at least a year,^11^ greater persistence with spaced induction protocols than with massed protocols, input specificity, associativity (both between presynaptic inputs and between pre- and postsynaptic cells), regulation by neuromodulators such as reinforcement signals, and ubiquitous expression at synapses throughout the nervous system (see also above-cited reviews). LTD shows many of these same properties, as expected of the bidirectional nature of memory mechanisms.^12^

LTP and LTD can be generated through several signal transduction pathways, triggered by both ionotropic and metabotropic receptor activation. Glutamate receptors have been of most interest as their ligand, the amino acid glutamate, is the predominant excitatory neurotransmitter in the mammalian nervous system. The most commonly studied form of LTP is that which is dependent on activation of the N-methyl-D-aspartate (NMDA) subtype of glutamate receptor. This receptor is of particular interest as it forms an ion channel, the opening of which is dependent on the degree of depolarization of the postsynaptic cell at the precise time that the neurotransmitter glutamate is binding to it. This makes the receptor/channel a “coincidence detector” that explains the LTP properties of input specificity and associativity.^7^ The input specificity arises because LTP is induced only at those synapses at which glutamate has bound to the NMDA receptor, while associativity arises from the need for multiple excitatory synapses to be co-active (i.e., associated in time and space) in order for there to be sufficient postsynaptic depolarisation to unblock the channel. NMDA receptor/channels are also permeable to calcium ions, well understood to be a critical initiator of the signalling cascades that lead to LTP induction. Remarkably, NMDA receptor activation and elevated intracellular calcium can also trigger LTD^12^; it is the spatiotemporal nature of the calcium signal, together with its overall amplitude as supported by other sources of calcium, that determines the direction of the synaptic change.^13^

Of course, while glutamate drives depolarization, this is tightly regulated by many factors. Most obvious here is gamma-aminobutyric acid (GABA)-mediated inhibition, which shapes patterns of depolarization at all levels from synapse to neuronal network. GABAergic inhibition is often considerable and may have to be alleviated for the induction of plasticity at excitatory synapses^14^ and various forms of learning.^15,16^ Excitatory inputs onto GABAergic interneurons may be up- or downregulated by LTP or LTD, thus regulating the likelihood of spiking in GABAergic cells and providing a mechanism for flexible feedback or feedforward inhibition onto excitatory cells.^17^ However, GABAergic synapses are themselves highly plastic structures,^15^ providing another mode by which inhibition can be molded by experience.^15,17^ While a thorough dissection of the roles of inhibitory plasticity are beyond the scope of this manuscript, these mechanisms are thought to maintain an excitation-inhibition balance in order to maintain stability and precise timing of neuronal firing.^15^

Additional levels of plasticity regulation come from a variety of sources. These include a plethora of neuromodulatory transmitter systems that can bias synapses in favour of either LTP or LTD independently of NMDA receptor activation,^18^ and the family of mechanisms collectively termed “metaplasticity”, in which neural activity at a given point in time elicits a persistent state of altered susceptibility to later plasticity.^19^ Popular models of neuronal function, such as the Bienenstock, Cooper and Munro model and its derivatives,^20^ entail a computational description of how the level of conjoint pre- and postsynaptic activity determines whether LTD or LTP is elicited. The models also include a metaplastic component in which susceptibility to LTP or LTD are regulated homeostatically by the recent history of neuronal activity. More thorough discussion of BCM theory and metaplasticity is available elsewhere.^19,20^

### Mechanisms of LTP persistence

Because of our particular interest on the mechanisms of memory longevity, we will focus here on this aspect of LTP. LTP persistence can be categorised into three phases, termed LTP1, 2 and 3^21^ after Racine et al.,^22^ but which the field more commonly refers to as early LTP (E-LTP) and late-LTP (L-LTP). E-LTP equates to Racine et al.’s LTP1 and refers to LTP that is independent of de novo protein synthesis. This form of LTP typically lasts only a few hours at most. L-LTP is protein synthesis-dependent and can be subdivided into LTP that is independent (LTP2) or dependent (LTP3) on transcription as well.^23^ LTP2 is possible because the synaptodendritic compartments contain protein synthesis machinery, and thus it is possible for protein synthesis from existing mRNA to be activated by synaptic signals in these compartments without the need for somatic transcription as a first step. Still, a sufficiently strong stimulation of excitatory afferents will engage transcription postsynaptically through activation of first constitutive transcription factors (e.g., cAMP response element binding protein [CREB], serum response factor, nuclear factor kappa B) and then downstream inducible transcription factors (e.g., zif268, c-fos, junB). Although LTP2 and LTP3 are generally studied in vitro, precluding easy analysis of the time course of the LTP phase they represent, in vivo studies have suggested that these mechanisms can support LTP lasting days, versus weeks or longer, respectively.^11,24^ Although a large number of genes and proteins have been identified as being altered in their expression following LTP, only a very few of these, e.g., brain derived neurotrophic factor, protein kinase Mζ, calcium/calmodulin-dependent protein kinase II, and activity-related cytoskeletal protein, have so far been implicated in the induction or maintenance of L-LTP.^25–28^

The protein synthesis-dependence of L-LTP enables a novel form of associative interaction between synaptic inputs. Thus, proteins synthesized in response to strong stimulation of one input pathway are able to be “captured” by a second converging input pathway and utilised to generate L-LTP even though that pathway had received only E-LTP generating stimulation. These data are explained by the synaptic tag and capture (STC) hypothesis, which posits that a weakly stimulated set of synapses sets a “tag”, yet to be fully defined mechanistically, that is able to sequester and utilise key proteins that are available in the vicinity, if they have been synthesized in response to stimulation of other synaptic inputs.^29^ These STC associative interactions can occur across intervals of hours,^30^ which extends the range of temporal contiguity needed for association well beyond the tens of milliseconds typical of Hebbian associativity. Interestingly, L-LTD, which is also protein synthesis-dependent, can undergo similar STC interactions. In fact, one or more of the proteins generated during L-LTP can promote L-LTD in neighboring synapses, and vice-versa, in a process that has been termed cross-tagging.^31^ STC may serve to bind together events that occur in temporal proximity to strongly stored, perhaps emotionally charged, experiences.

Additional support for the STC hypothesis comes from studies of a form of long-term synaptic plasticity in the marine snail *Aplysia californica* known as long-term facilitation (LTF). This form of plasticity occurs at synapses between sensory and motor neurons in *Aplysia* and underlies long-term sensitization, a form of non-associative memory in this animal.^32^ LTF can be induced in vitro by “training” sensorimotor cocultures with five spaced pulses of serotonin (5-HT),^33^ the monoaminergic transmitter that mediates sensitization.^34^ As is the case for L-LTP, both protein synthesis and gene transcription are required for LTF.^33^ Martin et al.^35^ demonstrated synapse-specific LTF through the use of cocultures having a single presynaptic sensory neuron and two postsynaptic motor neurons. When one of the two sensorimotor synaptic connections was selectively treated with five pulses of 5-HT (strong training), the trained synapse exhibited LTF, whereas the other synapse did not. But when the investigators paired delivery of a single pulse of 5-HT—which, by itself, induces only short-term facilitation—to one sensorimotor synapse with five pulses of 5-HT delivered to the other synapse, both synapses undergo LTF. Apparently, therefore, the weakly stimulated synapse can capture the LTF-inducing cell body products, whose synthesis is triggered by the strong training. This result implies that the single pulse of 5-HT causes tagging of a synapse, allowing it to capture plasticity-inducing molecules synthesized in the soma. The molecular identity of the tag that mediates synaptic capture for LTF is not yet known.

Returning to LTP, further evidence for a transcriptional role in L-LTP comes from studies of the epigenetic regulation in this form of synaptic plasticity. For example, histone deacetylases (HDACs) are potent negative regulators of gene expression, and HDAC inhibitors can convert E-LTP to L-LTP in vitro and promote long-term memory formation, particularly when given just prior to LTP induction or learning.^36^ Similarly, DNA methylation is a potent stabiliser of gene expression, and enhancing DNA methylation by inhibiting DNA methyltransferases or knocking out DNMT3a potently inhibits LTP,^37,38^ an effect that can be reversed by HDAC inhibition.^39^ Corresponding interactions between DNA methylation and HDAC inhibition were observed for the consolidation of fear conditioning in the amygdala.^39^ However, other studies suggest that the inhibitory role of DNA methylation in memory is not so clear-cut, as discussed below.

### Synaptic plasticity and memory hypothesis

Martin et al. have proposed several lines of evidence needed to confirm the crucial role of synaptic plasticity as a memory storage mechanism.^4^ These include: (1) detectability, in that learning should result in detectable changes in synaptic weight; (2) mimicry, whereby instituting those same synaptic weight changes for a given memory in a naïve animal should create the same memory; (3) anterograde intervention, whereby prevention of synaptic weight changes should prevent learning; and (4) retrograde intervention, whereby interference with the synaptic weight changes should erase the memory. Similar suggestions have been made by others.^40^ From these multiple approaches, the evidence supporting a role for synaptic plasticity in the storage and maintenance of memory has been converging to support the hypothesis. Synaptic plasticity is detectable in relevant brain structures after many forms of learning,^41,42^ and various forms of reversal learning induce a complementary reversal of synaptic plasticity that had been induced following the initial learning episode.^43–45^ By far and away the most evidence for the synaptic plasticity and memory hypothesis comes from anterograde intervention approaches using prior plasticity induction or pharmacological/genetic manipulations to block both LTP and learning.^4^ In contrast, there is very little evidence that mimicry is possible, with the closest attempt to date being the reinstatement of auditory fear conditioning following the reinstatement of LTP of specific auditory inputs to the amygdala.^45^ Perhaps the approach most pertinent to the theme of this paper, however, is retrograde intervention, which addresses the issue of whether altered synaptic weights maintain memory. Indeed, generation of LTP in the dentate gyrus post-training can cause amnesia for a previously stored spatial memory^46^, presumably by disrupting the differential weighting of synapses strengthened during learning and those that were not, while administration of zeta-inhibitory-peptide (ZIP) as well as over-expression of a dominant-negative PKMζ, treatments known to reverse LTP, cause memory loss across a range of tasks.^26,47,48^ Conversely, selective pharmacological blockade of LTP reversal (depotentiation) also blocks reversal learning in the Morris water maze.^49^ In a different approach, motor learning can be erased by optogenetically depotentiating the specific spines that were strengthened during learning.^50^ Also, amnesia for fear conditioning engineered by pairing footshocks with optogenetic stimulation of auditory pathways to the amygdala can be generated by depotentiating optogenetic stimulation and subsequently restored by potentiating stimulation delivered to the auditory pathways.^45^ Whether the memory per se in these studies was stored at the auditory pathway-lateral amygdala synapses is not entirely clear, as it is possible that the critical synaptic changes occurred elsewhere but could not be retrieved by under-strength auditory pathway activity. However, recent findings have lent even stronger support to the synapse plasticity basis of fear memory in the amygdala. For example, it has been shown that input specific LTP occurs in the amygdala during discriminative fear learning, and that depotentiating those same synapses causes memory loss.^44^ Moreover, when two distinct memories share overlapping neuronal ensembles in the amygdala, optogenetic potentiation or depotentiation of the synapses encoding one of those memories selectively affects only that memory, without changing the maintenance and recall of the second memory.^51^ Together these findings lend strong support to the synaptic plasticity and memory hypothesis.

### LTP and LTD in invertebrates

Neither LTP nor LTD is unique to the vertebrate nervous system; these two forms of synaptic plasticity are also expressed in invertebrate nervous systems. The first demonstration of NMDA receptor-dependent LTP at an invertebrate synapse was reported in *Aplysia*, where it was shown that either high-frequency, or paired pre-and postsynaptic (associative) stimulation can potentiate the synaptic connection between the sensory and motor neurons that mediate this animal’s defensive withdrawal reflex.^52,53^ In addition, LTD, induced by homosynaptic, low-frequency stimulation and dependent upon postsynaptic calcium, has also been reported for the *Aplysia* sensorimotor synapse.^54^ As is the case in the mammalian brain, both NMDA receptor-dependent and NMDA receptor-independent forms of LTP can be co-expressed in the central nervous system (CNS) of some invertebrates. Thus, in the central ganglia of the leech, repeated tetanic stimulation (at 25 Hz) induces NMDA receptor-dependent LTP at the synapse between the pressure (P) cells and the S cells. (The latter are interneurons whose activity contributes to reflexive whole-body shortening in the leech.) Tetanic stimulation also induces non-NMDA receptor-dependent LTP at the synapse between neurons responsive to touch (T cells) and the S cell.^55^ The T-to-S LTP requires activation of metabotropic glutamate receptors (mGluRs), voltage-dependent calcium channels, and protein kinase C.^56^ Interestingly, when the T-to-S LTP is blocked (through the use of an mGluR antagonist, for example), LTD is revealed in the neighbouring synapse between a nontetanized (control) T cell and the S cell; this heterosynaptic LTD is blocked by antagonists of NMDA receptors.^56^ In addition, low-frequency (1 Hz) stimulation for either 450 s or 900 s can induce LTD of the T-to-S cell synapse; however, whereas the LTD induced by 450 s of stimulation depends on NMDA receptor activation, the LTD induced by 900 s of stimulation does not. Instead, the 900 s low-frequency-induced LTD requires activation of endocannabinoid receptors.^57^

The vertical lobe, a learning-related structure in the brain of cephalopods, also expresses mechanistically distinct forms of LTP. The glutamatergic synapses between the neurons of the superior-frontal lobe, which is involved in sensory integration, and the amacrine cells of the vertical lobe exhibit two types of LTP in response to high-frequency stimulation of the superior-frontal lobe pathway. Induction of one form of LTP is due entirely to presynaptic mechanisms, whereas induction of the other form requires a postsynaptic influx of calcium, and therefore appears to depend on a Hebbian-type associative mechanism; curiously, however, NMDA receptors do not seem to mediate the latter type of LTP.^58^ The amacrine cells of the vertical lobe form cholinergic synapses with large efferent neurons that project out of the vertical lobe. The amacrine-to-large efferent neuron synapse does not exhibit LTP in the octopus, but does exhibit LTP in another cephalopod, the cuttlefish.^59^ Another intriguing contrast between the octopus and cuttlefish is that high-frequency stimulation does not potentiate the superior-frontal-lobe-to-amacrine-cell synapses in the cuttlefish. The functional significance of these differences in synaptic plasticity expressed in the two species is unknown at present. Finally, paired pre- and postsynaptic (Hebbian) stimulation can induce either LTP or LTD at synapses in the so-called mushroom body—a brain structure involved in olfactory learning and memory—in the locust, depending on whether the presynaptic action potential precedes or follows the postsynaptic action potential^60^; this phenomenon, known as spike-timing dependent plasticity (STDP), is also exhibited by synapses in the mammalian brain.^61^

As the above summary indicates, there are striking mechanistic similarities between LTP and LTD in vertebrates and invertebrates, which suggests that these two forms of synaptic plasticity have been evolutionarily highly conserved. There is also evolutionary conservation of the behavioral functions of LTP and LTD. For example, NMDA receptor-dependent LTP at the *Aplysia* sensorimotor synapse plays a critical role in classical conditioning of this animal’s defensive withdrawal reflex.^62,63^ Furthermore, the T-to-S and P-to-S neuronal pathways in the leech, which exhibit both LTP and LTD, are involved in several learning-related modifications of the shortening reflex, including habituation, dishabituation, and sensitization.^64^ In addition, LTP of the superior-frontal-lobe-to-amacrine-cell pathway is involved in long-term, associative memory in the octopus.^65^ Regarding potential roles for LTP learning and memory in insects, the mushroom body has been implicated in olfactory classical conditioning in these animals,^66^ and it seems likely that potentiation of synapses within the mushroom body, including NMDA receptor-dependent LTP^67^ mediates, at least in part, this form of associative learning. Finally, insects exhibit long-term habituation to prolonged exposure to an odor, and in *Drosophila* this non-associative memory is mediated by NMDA receptor-dependent LTP of input from local inhibitory interneurons—which release both GABA and glutamate—to neurons within the antennal lobe that project to the mushroom body.^68^

The above is not intended as an exhaustive review of LTP and LTD in invertebrates. Nonetheless, as should be clear, these two forms of synaptic plasticity are widely expressed by invertebrate nervous systems, and as well play important roles in invertebrate learning and memory.

### What is the mechanism by which long-term memories are maintained?

As noted previously, there is an overwhelming amount of evidence that synaptic plasticity is a fundamental mechanism contributing to memory storage. However, it is still an open question as to whether maintenance of those newly altered synaptic weights is necessary for maintaining the memory. Yes, experimentally induced LTP can last a very long time, but the same LTP is erasable by experiences associated with enriched environment exposure, a treatment which doesn’t necessarily erase hippocampus-dependent memory.^11^ Early studies using high resolution and repeated imaging of spines in the neocortex in vivo reported widely varying rates of spine turnover, ranging from 4% per month to 40% in 8 days,^69^ showing that spines vary in their durability. This variability of results may be a function of the methodology used to access the cortex for viewing, with the less obtrusive thinned skull approach yielding greater estimates of spine stability.^69^ However, even with the use of more intrusive cranial windows, spine stability could be enhanced by generating optogenetically specific patterns of activity in layer 5 cells.^70^ In basal dendrites of CA1 pyramidal cells, microendoscopy has revealed a relatively high rate of spine turnover, with a mean lifetime of 1–2 weeks.^71^ Although as noted above this estimate could be affected by the invasive nature of the imaging technique, the rapid rate of turnover compared to cortex is consistent with theories of the hippocampus as a temporary memory store, with longer term storage of explicit memories in neocortex.^72^ Indeed, results from thinned skull experiments in layer V neurons of mouse cortex suggest not only greater longevity of spines formed post-learning^73^ but also a strong correlation between the retention of motor learning and the proportion of new spines formed and maintained after acquisition of the motor task.^74^

An alternative view to the need for permanent synaptic change to store long-term memories is that individual synapses can continue to change weights, as long as the overall network circuitry is structured such that the correct output occurs in response to a given input. In other words, there may be multiple synaptic weight distributions that can correctly couple inputs with outputs. It may be an important feature of networks that, as new information is learned, synaptic weights can be updated to incorporate the new information while retaining the old information. This has been shown to be necessary in artificial network models that have extensively coupled layers of cells in the network,^75^ and presumably this needs to be able to update network connectivity during new learning is the reason why memories become labile upon retrieval and then undergo a period of reconsolidation to store the new weight settings.^76^ Even months after learning, synapses still seem to undergo NMDA receptor-dependent modifications, which are necessary to keep the memories intact.^77^

Although different from the view that memories are stored by fixing a set of synaptic weights in a circuit for the lifetime of the memory, this dynamical view of synaptic plasticity and memory does not contradict the idea that memory retention depends on the efficacy of synaptic connections among the relevant neurons in the circuit where memories are stored. Rather, it asserts that ongoing plasticity of the connections may be a fundamental feature of memory storage for animals living in a dynamic and stimulating environment.

It should be noted here that recent developments from the Tonegawa lab offer a somewhat different model of synaptic plasticity and learning, in which LTP of existing synaptic connections is required for recall of a memory rather than its storage.^78^ In this model, based on experiments discussed in greater detail in the next section,^42^ learning establishes specific connectivity patterns between cells of a memory circuit (“engram”) and it is these new connections, rather than the potentiation of existing synapses, that supports memory storage.^78^ Further strengthening of connectivity in the form of LTP, perhaps occurring through local STC and synaptic clustering mechanisms,^79^ thus provides a scalar quantity that governs the ease of memory retrieval. It is unclear whether the new connections established by learning involve de novo synaptogenesis or the conversion of silent synapses to an active state, or how either of these mechanisms might be impervious to protein synthesis inhibition (see below). Nevertheless, this model could still be viewed by believers of the synaptic plasticity and memory hypothesis as being in keeping with the notion that synapses at some point in the circuit are the critical units of memory storage.

### An alternative model: cell-intrinsic storage of memory

While the synaptic plasticity model of long-term memory storage currently predominates in neuroscience, there are nonetheless both theoretical arguments and experimental data against the idea that long-term memory resides in synapses with learning-altered weights. Thus, Gallistel and colleagues have argued that the hypothesis that the engram consists of altered synaptic conductances is fundamentally flawed because a model based on this hypothesis cannot plausibly encode number^80^ (but see ref. ^81^ for an alternative view). A summary of the arguments regarding why synaptic conductances are not good mechanisms for encoding number, which involves a critique of the notion that temporal contiguity—how closely two stimuli occur in time—is crucial for associative learning, is beyond the scope of this review. However, one of the general difficulties raised by Gallistel and others for the synaptic plasticity model is pertinent here, namely, that synaptic molecules in the adult brain are not stable. The majority of synaptic proteins, whether pre- or postsynaptic, have been shown to have half-lives of only 2–5 days,^82^ (but see ref. ^83^), although it isn’t necessarily critical that individual molecules last for the life of a memory.^84^ Additionally, as described above, optical studies in which individual spines in living adult brains are repeatedly imaged over extended time periods indicate that these postsynaptic structures can exhibit significant dynamism, with the amount of spine turnover varying according to brain region, neuronal subtype, spine size and, possibly, the specific imaging methodology used,^69^ as noted above.

An alternative posed to the synaptic plasticity model is cell-intrinsic memory storage mediated by thermodynamically stable molecules. Indeed, findings from several recent studies provide support for the idea that memory can be stored in a cell-intrinsic fashion. Thus, Chen et al.^85^ have reported that the long-term memory for behavioural sensitization in *Aplysia* can be reinstated by abbreviated training—training that is insufficient to induce long-term memory in naïve snails—following its disruption by reconsolidation blockade. This occurs despite the apparent elimination by reconsolidation blockade of the synaptic growth that commonly accompanies long-term sensitization in *Aplysia*. In a related study, Ryan et al.^42^ demonstrated that the long-term memory for contextual fear conditioning in mice can be induced by optogenetic stimulation of hippocampal neurons that were active during behavioural training, even though the mice had received post-training injections of a protein synthesis inhibitor, a treatment that caused retrograde amnesia and eliminated conditioning-induced hippocampal LTP as well, at least at potentiated perforant path-dentate gyrus synapses. (Whether changes at other synapses in the memory circuit remained impervious to the protein synthesis inhibitor is not yet known, as is the degree to which protein synthesis was abolished even at affected synapses.^86^)

Additional evidence in favour of the cell-intrinsic hypothesis of memory storage comes from a study by Johansson et al.,^87^ who investigated how cerebellar Purkinje cells acquire information about the temporal interval between two conditioning-related stimuli. Their training protocol consisted of paired stimulation of the parallel fibers and the climbing fibers, the conditioned stimulus (CS) and unconditioned stimulus (US), respectively. Paired CS-US stimulation produced a reduction in the number of spikes evoked in the Purkinje cell by the parallel fibers; this reduction is believed to be due, at least in part, to associative LTD of the parallel fiber-to-Purkinje-cell synaptic connections.^88^ In their study, Johansson et al. carried out training using different intervals between the CS and US. Their data indicated that the memory of the trained CS-US interval could not reside in either excitatory or inhibitory networks within the cerebellum, but, rather, must result from mechanisms intrinsic to the Purkinje cells.

### Role of DNA methylation and demethylation in memory consolidation and maintenance

The most likely candidate for a cell-intrinsic engram mechanism, first proposed by Holliday,^89^ is epigenetic storage of information, particularly DNA methylation. (This idea was adumbrated by Francis Crick in 1984.^90^) As suggested by Holliday,

“specific sites in the DNA of neurons required for memory can exist in alternative methylated or non-methylated states. The initial signal which is to be memorized, switches the DNA from a modified to an unmodified state, or vice versa. This changes the phenotype of the neuron, so that when the same signal is received it now responds by firing, that is, it sends an electrical signal to other neurons with which it is in contact. A neuron which had not received the initial signal would not respond.” (339)

Holliday pointed out, as did Crick before him, that such a mechanism is intrinsically quite stable: “any turnover of DNA by repair will almost invariably involve only one strand, and the short newly synthesized region will become methylated.” As a candidate for an engram mechanism, DNA methylation has, in addition to relative stability, the advantages of compactness and energy efficiency. Furthermore, given the number of methylation sites in the whole genome, this mechanism can potentially encode vast amounts of information. Another epigenetic mechanism considered by Holliday is modification of histones, the main proteins in chromatin, which may undergo chemical modification, particularly acetylation or deacetylation. But he pointed out that because histones are not covalently linked to DNA, “the state of acetylation will not provide the same stability as the covalent linkage of a methyl group to cytosine in DNA.” Holliday further emphasized that DNA methylation may not underlie the storage of all types of memory. For example, he noted that the DNA of *Drosophila* appeared to lack cytosine methylation, but fruit flies nevertheless exhibit long-term memory.^91^ Since the publication of Holliday’s paper, however, there have been reports of DNA methylation in *Drosophila*; this phenomenon appears to be associated primarily with development,^92^ but DNA methylation has now been documented in adult flies as well.^93^

Holliday’s hypothesis that DNA methylation might subserve memory has received striking confirmation during the past decade. Studies in mammals and invertebrates have documented roles for DNA methylation in the formation of a variety of forms of learning and memory.^94–96^ These studies have shown that inhibitors of DNA methyltransferase (DNMT) block the formation and/or consolidation of memory. In addition, extensive DNA methylation changes have been documented for hippocampal-dependent fear conditioning in the brains of mice and rats; these involve both hypermethylation and hypomethylation (see below) of genes.^97,98^ Moreover, the pattern of DNA methylation changes alters over time, with some patterns apparently associated with long-term maintenance of memory, because they occur weeks after training. Note that because DNA methylation is commonly associated with gene silencing,^99^ the late DNA hypermethylation observed in these studies suggests the persistence of some forms of memory requires the ongoing repression of one or more genes. Further support for this intriguing idea has come from two studies, one in rats^100^ and the other in *Aplysia*,^96^ which found that DNMT inhibitors disrupt well-consolidated memory.

DNA methylation was long regarded as a more-or-less irreversible epigenetic modification; if so then, although well suited for the regulation of development, DNA methylation would seem ill-suited to mediate the neurobiological plasticity underlying learning and memory. In addition, it is difficult to conceive of a general memory mechanism that relies solely on the downregulation of gene activity. It is now apparent, however, that alteration of DNA methylation is a more dynamic process than originally believed. In particular, it has been discovered that DNA can be actively demethylated. An initial step in the active demethylation of DNA is conversion of 5-methylcytosine to 5-hydroxymethylcytosine by the Ten-eleven translocation 1–3 (Tet1–3) family of DNA hydroxylases; this is followed by base excision repair.^101,102^ Recently, Tet proteins in the brain have been shown to play critical roles in a variety of different forms of learning and memory or learning-related synaptic plasticity.^95,103,104^ The discovery of active demethylation of DNA means that cytosines in the genes of neurons can function as on-off switches, and thus could in principle subserve a binary code. A fascinating question is whether the mnemonic machinery of the brain makes use of this binary code. If so, how does the code get read out with respect to the alteration of synaptic connectivity?

### The synapse specificity problem

Perhaps the strongest argument against the cell-intrinsic hypothesis of memory storage, whether by epigenetic changes or another mechanism, is the extensive evidence that LTP, as well as other forms of learning-related long-term synaptic plasticity,^35^ exhibit input (or synapse) specificity.^105^ As discussed above, the most widely accepted explanation for synapse specificity in synaptic plasticity is the STC hypothesis. In addition to the evidence that long-term synaptic plasticity involves synaptic tagging, there are also data indicating that synaptic tagging is crucial for the consolidation of long-term memories.^106^ Of course, the synaptic model of memory storage easily accommodates the phenomenon of synapse specificity. It would appear far more difficult to accommodate this phenomenon in a cell-intrinsic model. How could the nucleus of a neuron in the central nervous system, given epigenetic memory storage, for example, encode knowledge of which specific synapses, out of the thousands of synaptic connections made by the neuron, were strengthened during a specific learning experience? One possibility, although admittedly speculative, is that cell bodies of neurons within a specific neural circuit have available molecular, non-synaptic pathways whereby the neurons can directly exchange information regarding learning-related interactions with each other. Non-coding RNAs, particularly microRNAs (miRNAs) and PIWI-interacting RNAs (piRNAs) represent a potential mechanism for such a pathway.^107^ Non-coding RNAs (ncRNAs) are known to mediate learning-related epigenetic alterations in neurons.^107^ Moreover, exosomes containing miRNAs have been shown to be released from neurons and taken up by other recipient neurons via endocytosis.^108^ In addition, the release of exosomes, containing specific species of miRNAs in some cases, can be driven by neuronal activity.^109,110^ Another potential pathway for direct neuron-to-neuron transfer of ncRNAs are tunneling nanotubes,^111^ long cytoplasmic bridges between neurons that permit the interneuronal exchange of vesicles, organelles, and a variety of small molecules.

Thus, exchange of ncRNAs, via exosomes or tunneling nanotubes, could, in principle, mediate the communication among neuronal somata of information regarding neural activity, neural state, and, perhaps, neuronal identity. Such non-synaptic communication might be more common within the nervous system, and of greater functional significance, than currently appreciated. This idea receives support from recent dramatic discoveries about the activity regulated cytoskeletal-associated (Arc) protein. Arc, an immediate early gene product, has long been recognized as an important regulator of synaptic plasticity. The transcription of Arc is induced by synaptic activity; Arc mRNA is then transported to dendrites where it is locally translated.^112^ Synaptically localized Arc protein modulates the trafficking of AMPA-type receptors at synapses, thereby regulating both LTP and LTD. Unexpectedly, however, as recently shown by two groups, one working in mice^92^ and another in flies,^113^ the Arc protein has the capsid-like structure of retroviruses; moreover, like retroviruses it encapsulates RNA, specifically, Arc mRNA. Neural activity stimulates the release of Arc proteins, packaged in exosomes, from neurons, and the Arc-containing exosomes are then taken up by neighboring neurons. Importantly, the Arc mRNA can undergo translation within these neurons. At present, the function of the transferred Arc mRNA within the recipient neurons, particularly its potential role in learning and memory, is not known. Nor is it known whether the exosomal Arc proteins contain other species of RNA, including ncRNAs. Nonetheless, these surprising findings regarding Arc, hitherto viewed as a synaptic plasticity protein, reveal a previously unsuspected RNA-mediated thoroughfare connecting neurons, one whereby a neuron can, at least in theory, profoundly alter, in a more-or-less direct way, gene expression in its neighbors. It remains to be determined whether the interchange of RNA—mediated by Arc and possibly other retroviral-like proteins—among neighboring neurons endows the somata of those neurons with the capacity for recognizing one another’s respective role in prior episodes of learning. Also to be determined is how synaptic enhancement mediated by the non-synaptic exchange of RNA among neurons can preserve synaptic specificity (see below).

### Memory transfer via RNA

The strongest challenge to date to the synaptic plasticity hypothesis of memory storage comes from a recent study by Bédécarrats et al.,^114^ who reported successful transfer of memory from a trained to an untrained animal via RNA injection. In their experiments, which were performed on *Aplysia*, Bédécarrats and colleagues gave one group of animals training on two consecutive days with a series of electrical shocks delivered to their tails; the training induced long-term sensitization, a non-associative form of learning, in the animals. The sensitization was expressed as an enhancement of the siphon-withdrawal reflex (SWR) to a weak touch delivered 24 h after the last episode of tail shocks. A control group of untrained snails exhibited no reflex enhancement when tested at the same time. Following the behavioral tests, central ganglia were dissected out of the trained and untrained animals, and the total RNA was extracted from the ganglia. The RNA was then purified, after which the RNA from the trained animals (hereafter trained donor RNA) was injected intrahemocoelly into a group of untrained recipient snails, and the RNA from the untrained animals (hereafter untrained donor RNA) was injected into a second group of untrained recipients. When tested 24 h post-injection, the animals that received the trained donor RNA exhibited significant enhancement of the SWR, whereas the recipients of the untrained donor RNA did not. Importantly, the sensitizing effect of the trained donor RNA, like tail shock-induced long-term sensitization,^96^ depended on an epigenetic change, DNA methylation: when the RNA injection was followed immediately by an injection of the DNMT inhibitor RG108, the behavioral enhancement was blocked.

As well as behavioral evidence for the transfer of sensitization memory, the investigators obtained cellular evidence for memory transfer. Previously, it was shown in *Aplysia* that long-term sensitization is accompanied by persistent hyperexcitability of the central sensory neurons,^32^ as well as by long-term facilitation of sensorimotor synapses.^32,115^ Bédécarrats and colleagues observed that incubation in trained donor RNA for 24 h significantly enhanced the excitability of isolated sensory neurons in dissociated cell culture; sensory neurons similarly incubated in untrained donor RNA did not exhibit enhanced excitability. Moreover, the effect of the trained donor RNA was cell type-specific: treatment with the RNA from trained animals did not alter the excitability of isolated motor neurons. The trained donor RNA also induced long-term facilitation of a subset of sensorimotor synapses in dissociated cell culture, although the mean effect of the RNA on synaptic strength was not statistically significant.

The data of Bédécarrats et al.,^114^ which are difficult—perhaps impossible—to reconcile with the synaptic plasticity hypothesis, offer dramatic support for the idea that long-term memory is stored as RNA-induced epigenetic alterations. These results in *Aplysia* provide an intriguing parallel to reports in the 1960s of memory transfer in planarians and rats. But the field of memory transfer was plagued by failures of replication and inability to obtain the phenomenon,^116^ and eventually died out. The study by Bédécarrats et al.,^114^ however, suggests that the general judgement that memory transfer was a scientific dead end may have been premature. A revival of the enterprise of memory transfer, informed by modern knowledge regarding epigenetics and ncRNA, may well be in order. A major unanswered question raised by the original memory transfer studies and that of Bédécarrats et al.^114^ is how the RNA from trained donor animals escaped rapid degradation by RNases upon being injected into the recipients. A potential answer to this question comes from the discovery of a group of RNAs, called circular RNAs (circRNAs), that are highly resistant to endonucleases.^117^ Interestingly, a recent study has implicated circRNAs in the brain in synaptogenesis and synaptic plasticity.^118^

### Synaptic plasticity and epigenetics: a potential synthesis

Ramon y Cajal was the most prominent early proponent of the synaptic plasticity hypothesis of learning and memory. Cajal’s ideas have predominated in neuroscience during the last century; indeed, the discovery of LTP and LTD, and the subsequent appreciation of the role of these two forms of synaptic plasticity in learning and memory, could be regarded as the ultimate triumph of the Cajalian view. But another hypothesis regarding brain function has been lurking in the intellectual shadows of neuroscience, that of Camilio Golgi, Cajal’s bitter rival and co-winner with Cajal of the 1906 Nobel Prize in Physiology or Medicine.^119^

Golgi believed that the nervous system comprised a syncytium, a net of fused brain cells. From the perspective of the twenty-first century, Golgi’s idea looks increasingly attractive. As discussed above, we now know that neurons can communicate non-synaptically, exchanging chromatin-altering RNA via exosomes or tunneling nanotubes; in other words, the brain may be viewed, from this perspective, as a functional syncytium. Of course, one does not yet know how important such non-synaptic communication is for the overall operation of the brain; nonetheless, it may well be that a comprehensive neurobiological understanding of learning and memory will require an integration of the ideas of Cajal and Golgi.

What would such an integration look like? One possibility is that learning-related synaptic plasticity triggers epigenetic changes in the nuclei of neurons, and that the memory is maintained by coordinated, ongoing communication between the synapse and the nucleus. There are many candidates for retrograde, synapse-to-nucleus signaling; among these are neurotrophins, transcription factors, and transcriptional coactivators, including Jacob, the Abelson interacting protein-1 (Abi-1), the amyloid precursor protein intracellular domain-associated protein-1 (AIDA-1), the CREB-regulated transcriptional coactivator (CRTC1),^120^ and even the C-terminus of the NMDAR GluN1–1a subunit.^121^ In addition, there are a variety of ncRNAs localized to dendrites, and these can be transported to the nucleus, where they can induce epigenetic changes.^117,122^ With respect to nucleus-to-synapse signaling, anterograde transport of mRNA from the nucleus to synaptic sites, where it can undergo local translation, is well known^123^; more recently, there is evidence that ncRNAs may also be transported from the neuronal cell nucleus to dendritic compartments.^124^ In a recently proposed alternative model, after learning there is an epigenetically mediated transcription repression and, presumably, reduced nucleus to synapse signaling (a “maintenance transcriptome”). According to this idea, the transcriptional repression maintains memory by preventing learning-induced neuronal plasticity from being readily overwritten by new experience-related plasticity.^125^

A more radical view is that long-term memory is entirely stored as epigenetic, or other^126^ changes within the nucleus. According to this view, synaptic changes are the mechanism whereby stored nuclear memories are expressed. This idea would account for recent data in *Aplysia* that indicate that at least some memories can persist in the absence of learning-induced synaptic alterations, following either reconsolidation blockade^85^ or post-training inhibition of protein synthesis.^96^ Moreover, the hypothesis of a strictly nuclear storage mechanism for memory could also accommodate the demonstration of memory transfer by RNA.^114^ Of course, acceptance of this hypothesis requires an explanation for the daunting problem of how the changes in the epitype of a neuron can faithfully maintain a record of the learning-induced changes in the thousands of synaptic connections maintained by the neuron, and no such explanation is presently at hand. Ultimately, determining which, if any, of these cell-intrinsic models of memory storage is correct will probably require an understanding of the extent and functional significance of the recently discovered transneuronal exchange of RNA.^92,113^

Finally, it could well be that synaptic-specific plasticity and cell-wide intrinsic plasticity mechanisms both play critical roles in memory storage and maintenance.^127^ The extent to which one predominates may depend on the nature of the memory and the circuit that is responsible for it. Sensorimotor reflex learning, as well as non-associative forms of learning, including habituation, sensitization, and, possibly, classical conditioning, may work perfectly, perhaps optimally, through cell-intrinsic mechanisms. More complex forms of learning, involving complex circuits in which thousands of synapses made by a neuron must be manipulated individually, or in small clusters,^79^ may require more local changes, and certainly in mammalian systems, the two mechanisms can converge. Cells with higher excitability have an advantage, both in generating synaptic plasticity and in being involved in memory storage, compared to their less active neighbors. Such effects have led to the memory allocation hypothesis,^6^ as well as the understanding of how young adult-born neurons in the hippocampus, which are unusually excitable and have a lower threshold for LTP compared to more mature cells, can play particular roles in memory storage and maintenance.^128^

## Conclusion

It has been almost half a century since the seminal discovery of LTP was reported by Bliss and Lømo.^129^ In the intervening time, neurobiologists have identified the mechanism that underlies this form of persistent, activity-dependent synaptic change—activation of the NMDA receptor—and shown that LTP mediates various forms of learning and memory, both in mammals and invertebrates.^9^ The establishment of these facts, which have confirmed the ideas of Cajal^119^ and Hebb,^1^ has involved a monumental effort by an army of researchers and represents one of the triumphs of modern neuroscience. Nonetheless, much work remains to be done. Whether or not memory is necessarily stored at synapses is still unclear.^80^ Moreover, recent discoveries indicating the importance of epigenetic changes^39,94,98,100^ and ncRNA in memory have yet to be fully incorporated into the synaptic plasticity hypothesis. Finally, the challenge of reconciling the synaptic plasticity hypothesis with the new demonstrations of intercellular transfer of RNA^92,113^ and of memory transfer by RNA^114^ must be met. The next 50 years will indeed be busy ones.
